# Irisin Increases Sirtuin 1 to Improve Glucocorticoid-Induced Sarcopenia and Mitochondrial Dysfunction

**DOI:** 10.3390/cells14211675

**Published:** 2025-10-27

**Authors:** Hongwei Shi, Wen Sun, Xiaoyuan Cao, Xuepeng Fan, Wenjuan Xie, Xiaojing Hao, Simiao Wang, Jiayin Lu, Yi Yan, Xiaomao Luo, Yanjun Dong, Haidong Wang, Juan Wang

**Affiliations:** 1 College of Veterinary Medicine, Shanxi Agricultural University, Jinzhong 030801, China; shw18434752340@163.com (H.S.); lujiayin2013@163.com (J.L.); yanyi@sxau.edu.cn (Y.Y.); lionelphilip@126.com (X.L.); 2 Shanghai General Hosptial, Shanghai Jiaotong University School of Medicine, Shanghai 200080, China; sunwensj@sjtu.edu.cn; 3 College of Veterinary Medicine, China Agricultural University, Beijing 100091, China; ii09657372@163.com (X.H.); yanjund@cau.edu.cn (Y.D.)

**Keywords:** irisin, sirtuin 1, glucocorticoids, muscle atrophy, mitochondrial dysfunction

## Abstract

**Highlights:**

**What are the main findings?**

- During the progression of glucocorticoid-induced metabolic sarcopenia, FNDC5/irisin levels are reduced, whereas supplementation of FNDC5/irisin alleviates glucocorticoid-induced metabolic sarcopenia.

**What is the implication of the main finding?**

- FNDC5/irisin ameliorates glucocorticoid-induced muscle protein metabolic imbalance via activation of sirtuin 1, thereby restoring muscle protein homeostasis.

**Abstract:**

Sarcopenia, characterized by progressive skeletal muscle mass, strength, and functional loss, imposes a substantial global health burden. Irisin, a myokine derived from fibronectin type III domain-containing protein 5 (FNDC5), is critical for muscle health. Here, we investigate its role in mitigating glucocorticoid-induced sarcopenia using a mouse and C2C12 myotubes model. We quantified FNDC5/irisin levels in skeletal muscle and plasma and assessed muscle function (body weight, grip strength, wire-hanging, and locomotor activity), histology, and mitochondrial features following irisin administration to dexamethasone-treated mice. Western blot analyzed synthesis/hydrolysis regulators, apoptosis markers, and mitochondrial regulators in mouse muscle tissues and C2C12 myotubes. The results show that FNDC5/irisin was significantly downregulated in sarcopenic mice and atrophic C2C12 myotubes; exogenous irisin rescued muscle mass loss and functional impairment, improving body weight, muscle mass, grip strength, and mobility. Mechanistically, irisin bound SIRT1 with −12.7 kcal/mol affinity, activating a deacetylation cascade that suppressed FoxO3a transcriptional activity (attenuating proteasomal degradation) and enhanced mTORC1-mediated protein synthesis in C2C12 myotubes. Additionally, irisin potentiated PGC-1α signaling in mouse myocytes, promoting mitochondrial biogenesis and restoring contractile function in dystrophic fibers. Collectively, these findings demonstrate irisin alleviates glucocorticoid-induced muscle atrophy via SIRT1-dependent pathways, rebalancing muscle physiology and systemic energy homeostasis. This highlights irisin-based therapeutics as a promising exercise surrogate for sarcopenia management, offering novel clinical avenues.

## 1. Introduction

As one of the largest organs in the human body and a primary protein reservoir, skeletal muscle plays a vital role in locomotion and metabolic regulation [1]. However, muscle integrity is frequently compromised by protein homeostasis imbalance, exemplified by sarcopenia-a pathological condition characterized by significant loss of skeletal muscle mass and strength. Sarcopenia arises from multifactorial aetiologies, including musculoskeletal trauma, ageing, diabetes mellitus, and neoplastic diseases [2,3], leading to severe reductions in patient independence and quality of life, while substantially increasing direct and indirect healthcare costs associated with disability. Glucocorticoids (GCs), widely prescribed for their anti-inflammatory and immunosuppressive properties [4], are a known aetiological factor for sarcopenia when administered chronically or at supraphysiological doses [5]. This condition may also manifest during endogenous GC overproduction under pathological conditions [6]. Clinical reports indicate a 60% incidence of glucocorticoid-induced myopathy [7]. Synthetic GCs, exemplified by dexamethasone (Dex), induce skeletal muscle atrophy through dual mechanisms: (1) suppression of muscle protein synthesis via inhibition of the Akt/mTOR signaling pathway [8], and (2) acceleration of proteolytic catabolism mediated by the ubiquitin-proteasome system (UPS). The latter process is orchestrated by forkhead box (FoxO) transcription factors, which activate two muscle-specific E3 ubiquitin ligases—muscle RING finger 1 (MuRF-1) and atrogin-1 (also known as MAFbx)—to drive muscle wasting [9]. While these pathogenic mechanisms have been extensively characterized, the molecular regulators that balance catabolic and anabolic pathways during GC-induced atrophy remain poorly understood, representing a critical knowledge gap in developing targeted therapies.

Mitochondrial dysfunction serves as a critical aetiological factor in muscle atrophy induced by disuse and pathological conditions [10]. Peroxisome proliferator-activated receptor γ coactivator 1α (PGC-1α), a master regulator of mitochondrial biogenesis, exhibits downregulated signalling in skeletal muscle mitochondrial dysfunction. Notably, activation of the PGC-1α pathway ameliorates muscle strength deficits and protein metabolism dysregulation [11]. Sirtuin 1 (SIRT1), a member of the evolutionarily conserved NAD+-dependent histone deacetylase family [12], plays a pivotal role in skeletal muscle remodelling. SIRT1 directly deacetylates and activates PGC-1α, thereby stimulating mitochondrial biogenesis in muscle tissues [13]. This mechanistic linkage positions SIRT1 as a promising therapeutic target for addressing muscle dysfunction.

To date, physical activity remains the most clinically validated intervention for sarcopenia; however, its implementation is constrained in elderly and mobility-impaired populations. This limitation has spurred intense interest in developing exercise mimetics as therapeutic alternatives for sarcopenia [14]. Irisin, a myokine secreted by myofibres during exercise-induced proteolytic cleavage of fibronectin type III domain-containing protein 5 (FNDC5) [15], plays a critical role in muscle regeneration and homeostasis. Beyond promoting post-injury repair, irisin enhances metabolic regulation and muscular strength, establishing its status as a key mechanotransduction molecule bridging physical activity and metabolic health [16]. Recent evidence demonstrates that irisin improves denervation-induced muscle injury by activating the IL-6 signalling pathway to enhance myogenesis and myoblast fusion, while simultaneously stimulating ERK1/2-dependent protein synthesis [17]. These findings position Irisin as a promising therapeutic target for sarcopenia intervention.

In this study, we investigated the alterations of FNDC5/irisin in GC-induced sarcopenia and evaluated whether irisin-based intervention could ameliorate GC-associated muscle atrophy and mitochondrial dysfunction. Notably, our results demonstrated that protein levels of FNDC5/irisin in both skeletal muscle and circulation were significantly downregulated during muscle wasting. Intraperitoneal injection administration of recombinant irisin protein attenuated GC-induced muscle atrophy and mitochondrial abnormalities in murine models. Collectively, these findings establish irisin as a promising exercise mimetic alternative for sarcopenia treatment, targeting pathological muscle loss and functional decline.

## 2. Materials and Methods

### 2.1. Animals

Male C57BL/6J mice (8 weeks old) were housed under controlled conditions (temperature: 22 ± 3 °C; humidity: 40–50%; 12 h light-dark cycle) with ad libitum access to food and water. Animals were randomly assigned to five groups (*n* = 6 per group) and administered treatments as follows: (i) Control group: vehicle (PEG300 solution, i.p.); (ii) Irisin group: The irisin stock solution was prepared using sterile physiological saline as the solvent at a concentration of 500 μg/mL (2.5 mg/kg, i.p.); (iii) Dex group: The formulation of the dexamethasone stock solution comprises PEG300 at a concentration of 2.5 mg/mL (25 mg/kg, i.p.); (iv) Dex + Irisin group: dexamethasone (25 mg/kg, i.p.) + irisin (2.5 mg/kg, i.p.); (v) Dex + Irisin + EX-527 (SIRT1 inhibiting drug) group: dexamethasone (25 mg/kg, i.p.) + Irisin (2.5 mg/kg, i.p.) + EX-527 (10 mg/kg, i.p.). Based on previous studies [16], Irisin was administered three times weekly, while dexamethasone and EX-527 were administered daily, over a total duration of 14 consecutive days. Body weight was recorded daily. Samples of blood, gastrocnemius (GAS), and tibialis anterior (TA) muscles were collected at 24 h following the last injection. Upon collection, the muscle tissues were immediately weighed and photographed, followed by fixation in 4% paraformaldehyde or flash-freezing in liquid nitrogen for subsequent analysis. All experimental manipulations were carried out in compliance with protocols authorized by the Animal Care Committee of Shanxi Agricultural University (SXAU-EAW2024M.ID.011005320).

### 2.2. Cell Culture and Differentiation

The mouse C2C12 myoblast cell line was acquired from the American Type Tissue Culture Collection (ATCC). Cells were maintained in high-glucose Dulbecco’s Modified Eagle Medium (BI, Beit HaEmek, Israel) supplemented with 10% fetal bovine serum (Sciencell, Carlsbad, CA, USA) at 37 °C in a humidified 5% CO_2_ atmosphere. Upon reaching 70% confluency, the growth medium was replaced with a differentiation medium consisting of DMEM and 2% horse serum to induce myotube formation. Cultures were maintained under this condition for 4 days, with medium replacement daily, to induce multinucleated myotube formation. To induce myotube atrophy, fully differentiated myotubes were treated with 50 μM Dex (MCE, Monmouth Junction, NJ, USA) for 24 h. R-irisin (Phoenix Pharmaceuticals, Burlingame, CA, USA) and EX-527 (MCE, Monmouth Junction, NJ, USA) were administered concomitantly with Dex during the atrophy induction phase.

### 2.3. Cell Viability Assay

C2C12 myotubes, fully differentiated following seeding at an initial density of 1 × 10^4^ cells per well in 96-well plates, were subjected to a 24 h treatment with 50 μM dexamethasone (Dex) and varying concentrations of R-irisin (250, 500, 750, and 1000 ng/mL). Cell viability was subsequently assessed using a Cell Counting Kit-8 (CCK-8; YEASEN, Shanghai, China). Briefly, 10 μL of CCK-8 reagent was added to each well, followed by a 2 h incubation at 37 °C. The absorbance was then measured at 450 nm using a microplate reader (Molecular Devices, San Jose, CA, USA). The percentage of cell viability was calculated relative to the untreated control group using the formula: Cell Viability (%) = (As − A0)/(Ac − A0) × 100%, where As, A0, and Ac were the absorptions of test sample, back-ground, and control (DMSO), respectively.

### 2.4. Cellular Immunofluorescence Staining

Following fixation with 4% paraformaldehyde (15–20 min) and permeabilization with 0.1% Triton X-100 (10 min), the cells were blocked for 30 min at room temperature using 5% goat serum (Solarbio, Beijing, China). Subsequently, the cells were incubated overnight at 4 °C with a primary antibody targeting myosin heavy chain (MyHC). After extensive washing, a species-matched Alexa Fluor-conjugated secondary antibody was applied for 1 h at 37 °C. Nuclei were visualized by counterstaining with DAPI for 10 min at room temperature. Fluorescence images were acquired with a Eclipse Ts2R microscope (Nikon, Yokohama, Japan) and processed using the NIS-Elements software (v4.20).

### 2.5. Tissue Section Preparation and Hematoxylin and Eosin Stain (H&E)

Muscle tissues were fixed in 4% paraformaldehyde for 24 h, followed by graded ethanol dehydration and xylene clearing. Paraffin-embedded tissues were sectioned into 0.8 μm-thick slices using an automated rotary microtome (LEICA, Hesse, Germany). The sections were deparaffinized with xylene, rehydrated through graded ethanol series, and stained with haematoxylin and eosin. After mounting with neutral balsam (Solarbio, Beijing, China) images were captured and processed using a microscope (Nikon, Yokohama, Japan) equipped with NIS-Elements imaging software.

### 2.6. Western Blot Analyses

Protein extracts were prepared from muscle tissues or myotubes by homogenization in cold RIPA buffer (Beyotime Biotechnology, Shanghai, China) containing a cocktail of protease and phosphatase inhibitors. Following lysis on ice for 30 min and centrifugation at 13,000× *g* for 5 min at 4 °C, the supernatant was collected. Protein concentration was determined using a BCA assay kit (Beyotime, Shanghai, China). Equal amounts of protein were then resolved by SDS-PAGE and electrophoretically transferred onto PVDF membranes (Millipore, Burlington, MA, USA). The membranes were blocked with 5% skimmed milk in TBST for 1 h at room temperature before being incubated with primary antibodies overnight at 4 °C. After washing, the membranes were probed with appropriate HRP-conjugated secondary antibodies for 1 h at 37 °C. Signal detection was performed using enhanced chemiluminescence reagents (ABBKINE, Wuhan, China), and band intensities were captured with a ChemiDoc XRS+ imaging system (Bio-Rad, Hercules, CA, USA). Quantitative analysis was conducted using ImageJ software (v1.47, National Institutes of Health, Bethesda, MD, USA) after normalizing the band intensities to the endogenous control GAPDH.

### 2.7. Immunoprecipitation

To distinguish the acetylated form of PGC-1α from its total protein levels, we conducted immunoprecipitation (IP) followed by Western blotting. In this procedure, cell lysates were incubated with anti-PGC-1α antibody and A/G agarose beads (Santa Cruz Biotechnology, Santa Cruz, CA, USA) overnight at 4 °C under constant rotation. The beads were then pelleted and washed extensively with cold RIPA buffer. Bound proteins were eluted by heating in 5×SDS sample buffer and subjected to SDS-PAGE. Immunoblotting was subsequently carried out, employing an antibody against acetylated lysine to detect PGC-1α acetylation, with total PGC-1α serving as the internal control.

### 2.8. Cellular Mitochondrial Membrane Potential Assay

C2C12 myoblasts were seeded in confocal culture dishes and allowed to reach full differentiation. Differentiated myotubes were with dexamethasone (50 μM), R-irisin (1000 ng/mL), and the SIRT1 inhibitor EX-527 (20 μM) for 24 h. According to the manufacturer’s instructions, live cells were stained with JC-1 fluorescent dye (Beyotime Biotechnology, C2003S, China) at 37 °C for 20 min to visualize the level of mitochondrial membrane potential. Mitochondrial membrane potential was visualized by capturing fluorescence images using a confocal laser scanning microscopy (CLSM) system (Olympus, Yokohama, Japan).

### 2.9. Determination of ATP Content

ATP concentrations were measured following the instructions of a commercial detection kit (Beyotime Biotechnology, S0026, China). Muscle samples were homogenized in ice-cold lysis buffer and centrifuged (12,000× *g*, 5 min, 4 °C). The supernatant was then incubated with the ATP detection reagent, and luminescence was recorded on a FilterMax F5 microplate reader (Molecular Devices, San Jose, CA, USA). Sample ATP levels were interpolated from a standard curve.

### 2.10. Sirtuin1 Deacetylase Activity

SIRT1 deacetylase activity was quantified using a fluorometric assay kit (Elabscience^®^, Wuhan, China) following the manufacturer’s instructions. Briefly, 1 × 10^6^ cells were homogenized in 0.2 mL physiological saline, centrifuged at 12,000× *g* for 5 min at 4 °C, and the supernatant was collected. The cell lysate was incubated with reaction buffer in a 96-well plate, and fluorescence intensity was monitored at excitation/emission wavelengths of 340/440 nm using a microplate reader (Molecular Devices, San Jose, CA, USA). Enzyme activity was normalized to total protein content.

### 2.11. Cellular Reactive Oxygen Species (ROS) Assay

Intracellular ROS levels were assessed using a commercially available ROS detection assay kit (Beyotime Biotechnology, Shanghai, China). C2C12 myoblasts were seeded in sterile cell culture dishes and allowed to reach full differentiation. Differentiated myotubes were incubated with the fluorescent probe DCFH-DA (dichlorofluorescin diacetate) at 37 °C for 20 min. Fluorescence intensity was quantified using a fluorescence microscope (Nikon, Yokohama, Japan) equipped with appropriate excitation/emission filters (Ex/Em = 488/525 nm).

### 2.12. Enzyme-Linked Immunosorbent Assay for Mouse Irisin

The concentration of irisin in plasma and cell supernatants from each mouse group was quantified using a species-specific enzyme-linked immunosorbent assay (ELISA) kit (Elabscience^®^, Wuhan, China) in strict accordance with the manufacturer’s instructions.

### 2.13. Skeletal Muscle Function Exploration

Exhaustion Time and Distance. Following a 2-day acclimation period, running performance was assessed as previously described [18], Mice ran on a treadmill (SANS Biotechnology, Nanjing, China) with a 13% incline. The speed was increased by 5 cm/s every 2 min until exhaustion, which was designated when a mouse’s hindlimbs remained on the electric grid for >10 s. The total time to exhaustion and running distance were recorded.

Limb Grip Strength. Forelimb and hindlimb strength was measured using a dynamometer (SANS Biotechnology, Nanjing, China). Mice were allowed to grasp a horizontal grid and were pulled backward ten times. The peak force (in Newtons) applied before the mouse lost its grip was recorded for each trial. The average of ten measurements was normalized to the animal’s body weight.

Inverted Grid Test. Motor coordination and endurance were evaluated by placing mice on a wire grid, which was then inverted over a 2 s period. The grid was held 40–50 cm above a soft surface. The latency to fall was recorded as the hanging time.

### 2.14. Protein Synthesis by In Vitro SUnSET

The SUnSET assay was employed to assess the global protein synthesis rate. Briefly, puromycin (1 μg/mL) was administered to the culture medium 10 min before cell harvesting. The resulting cell extracts were then subjected to Western blot analysis using an anti-puromycin antibody (12D10).

### 2.15. Observations on Mitochondria in Gastrocnemius Muscle

Ultrastructural analysis of muscle tissue was performed by transmission electron microscopy (FEI, Hillsboro, OR, USA). Briefly, samples were fixed in 1% glutaraldehyde, sectioned, and mounted on copper grids for observation under the microscope equipped with a high-resolution camera.

### 2.16. Flow Cytometry Detection of Apoptosis

Cell apoptosis was assessed with an Annexin V-FITC/PI detection kit (ABBKINE, Wuhan, China) per the manufacturer’s protocol. In brief, 1 × 10^5^ cells per sample were resuspended in binding buffer and stained with Annexin V-FITC and propidium iodide (PI) for 15 min at room temperature in the dark. Annexin V is a calcium-dependent phospholipid-binding protein that specifically recognizes and binds to phosphatidylserine (PS) externally exposed on the cell membrane. PI, a nucleic acid-binding dye, enters the cell and binds to nuclear DNA during the late stage of apoptosis when the cell membrane integrity is disrupted. The samples were then immediately analyzed on a BD LSRFortessa™ flow cytometer (Becton, Dickinson and Company, Franklin Lakes, NJ, USA), configuring the FITC (Ex/Em: 488/520 nm) and PI (Ex/Em: 535/615 nm) channels. The resulting data were processed using FlowJo software (v10.8.1, BD Biosciences, Franklin Lakes, NJ, USA).

### 2.17. Statistical Analysis

All the statistical data are presented as the mean ± standard error of the mean (SEM). Comparisons between two groups were performed using a two-tailed Student’s *t*-test, and comparisons among multiple groups were performed using one-way analysis of variance (ANOVA) with Tukey’s post hoc test. A *p* value of less than 0.05 (*p* < 0.05) was considered to indicate statistical significance. All the data were drawn using GraphPad Prism 8.3.

## 3. Results

### 3.1. FNDC5/Irisin Levels Decrease During Glucocorticoid-Induced Muscle Atrophy

**Figure 1:**
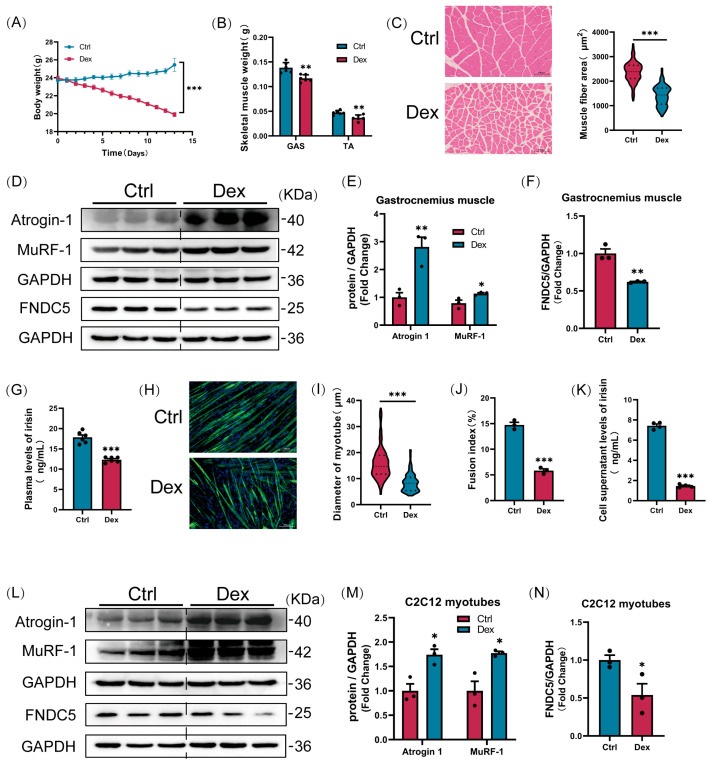
FNDC5/irisin levels decrease during glucocorticoid-induced muscle atrophy. (**A**) Body weight changes in control and Dex-treated mice (*n* = 6 per group). (**B**) Gastrocnemius (Gast) and tibialis anterior (TA) muscle mass analysis (*n* = 6 per group). (**C**) Representative haematoxylin and eosin (H&E) staining of myofibre cross-sections in Gast muscles (scale bar = 100 μm). (**D**–**F**) Western blot analysis of atrogin-1, MuRF-1, and FNDC5 protein levels in Gast tissues from control and Dex-treated mice (*n* = 3 per group). (**G**) Plasma irisin concentrations measured by ELISA in control and Dex-treated mice (*n* = 6 per group). (**H**–**J**) Analysis of myosin heavy chain (MyHC) immunofluorescence staining, myotube diameter, and myotube fusion index in C2C12 myotubes from the control and dexamethasone (Dex) groups. (scale bar = 100 μm, *n* = 3 per group). (**K**) Irisin levels in C2C12 myotube cultures quantified by ELISA (control vs. Dex: *n* = 4 per group). (**L**–**N**) Western blot validation of atrogin-1, MuRF-1, and FNDC5 expression in C2C12 myotubes under control and Dex conditions (*n* = 3 per group). Protein band densities were normalized to GAPDH. The data are presented as mean ± SEM;The symbol “•” denotes biological replicates; * *p* < 0.05, ** *p* < 0.01, *** *p* < 0.001.

To investigate the role of FNDC5/irisin in glucocorticoid-induced muscle atrophy, we employed Dex, a synthetic glucocorticoid analogue, to establish a well-characterized murine model of muscle wasting. Dex administration caused a marked reduction in body weight over the treatment period (Figure 1A). Consistent with systemic wasting, Dex treatment resulted in significant decreases in gastrocnemius and tibialis anterior muscle weights (Figure 1B) and reduced myofibre cross-sectional area in the gastrocnemius (Figure 1C). Notably, Western blot analysis confirmed elevated expression of atrophy-related ubiquitin ligases Atrogin-1 and MuRF-1 in Dex-treated muscles (Figure 1D,E). These data confirm that we successfully reproduced previously reported models of dexamethasone-induced muscle atrophy [19,20].

In parallel, FNDC5 protein levels in the gastrocnemius were significantly decreased following Dex exposure (Figure 1D,F). Strikingly, circulating irisin levels in plasma were also markedly reduced (Figure 1G). To translate these findings in vitro, we differentiated C2C12 myoblasts into myotubes and treated them with Dex. This treatment promoted myotube atrophy, manifested by decreased myotube diameter/fusion index (Figure 1H–J) and increased Atrogin-1/MuRF-1 expression (Figure 1L,M). Consistent with in vivo data, FNDC5 protein levels in Dex-treated myotubes were significantly diminished within 24 h (Figure 1N), accompanied by a sharp decline in secreted irisin levels in culture supernatants (Figure 1K). These observations suggest that suppression of the FNDC5/irisin axis may contribute to glucocorticoid-driven muscle wasting.

### 3.2. R-Irisin Protects C2C12 Myotubes Against Dexamethasone-Induced Atrophy

**Figure 2:**
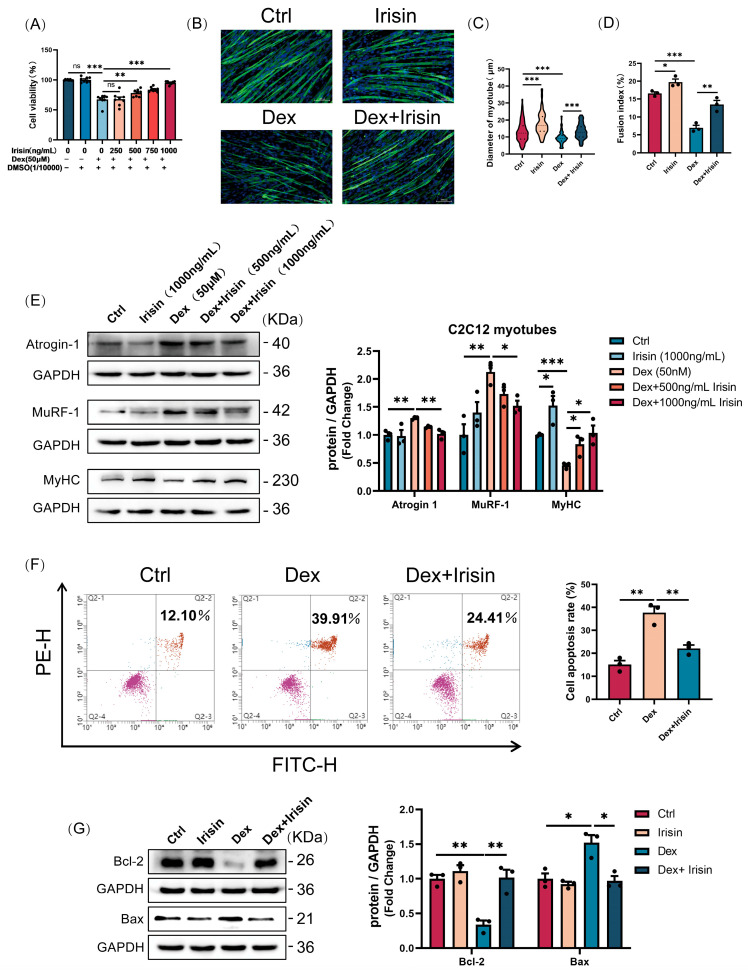
R-irisin protects C2C12 myotubes against dexamethasone-induced atrophy. (**A**) Cell viability of C2C12 myotubes treated with Dex and different concentrations of irisin (*n* = 8 per group). (**B**–**D**) Quantitative analysis of MHC immunofluorescence staining, myotube diameter, and myotube fusion index. (bars = 100 μm, *n* = 3 per group). (**E**) Western blot analysis of atrophy-related proteins (Atrogin-1, MuRF-1) and FNDC5 in Dex- and R-irisin-treated myotubes (*n* = 3 per group). (**F**) Flow cytometric analysis of apoptosis via Annexin V-FITC/PI co-staining (*n* = 3 per group). (**G**) Western blot validation of apoptosis regulators Bcl-2 and Bax expression (*n* = 3 per group). Protein band densities were normalized to GAPDH. The data are presented as mean ± SEM; The symbol “•” denotes biological replicates; ^ns^ *p* > 0.05, * *p* < 0.05, ** *p* < 0.01, *** *p* < 0.001.

Given the observed downregulation of FNDC5/irisin in the Dex-induced muscle atrophy model, we investigated whether irisin reconstitution could rescue Dex-mediated myopathic phenotypes. In C2C12 myotubes, R-irisin administration dose-dependently reversed Dex-induced reductions in myotube viability (Figure 2A). Similarly, R-irisin treatment effectively prevented Dex-induced myotube atrophy (Figure 2B,C). Furthermore, even at the baseline level, the myotube diameter and fusion index were significantly increased following R-irisin exposure (Figure 2B–D). Western blot analyses revealed that R-irisin administration suppressed Dex-induced upregulation of atrophy markers Atrogin-1 and MuRF-1 (Figure 2E). Crucially, R-irisin robustly restored myosin heavy chain (MyHC) protein levels—a hallmark of myotube formation—in Dex-treated myotubes in a dose-dependent manner (Figure 2E). Strikingly, R-irisin (1 μg/mL) significantly enhanced baseline MyHC expression in control myotubes (Figure 2E), suggesting intrinsic myogenic properties. Furthermore, R-irisin demonstrated potent anti-apoptotic effects, markedly attenuating Dex-induced apoptosis in myotubes (Figure 2F,G). These findings collectively indicate that R-irisin functions as a pro-myogenic factor, promoting myotube hypertrophy while rescuing Dex-mediated atrophy and apoptotic responses.

### 3.3. R-Irisin Alleviated Dex-Induced Sarcopenia in Mice

**Figure 3:**
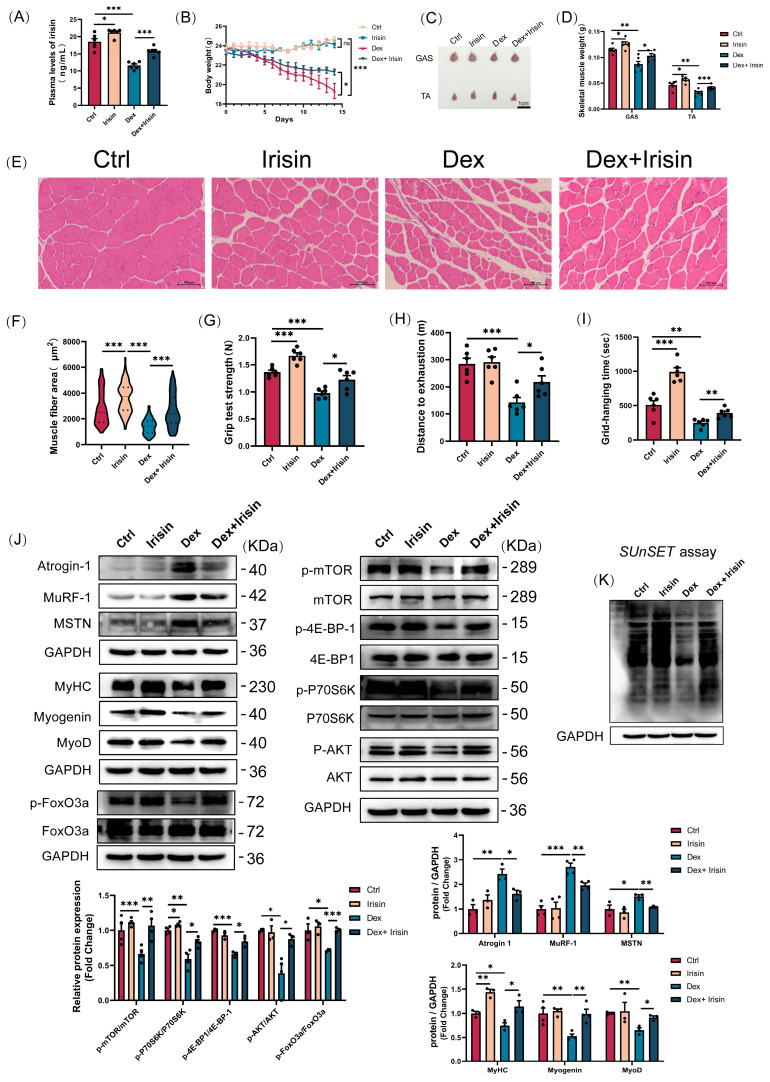
R-irisin reverses Dex-induced sarcopenia in mice. A murine model of muscle atrophy was induced using a relatively high dose of dexamethasone (25 mg/kg bw, i.p. 14 days) and treated with irisin (2.5 mg/kg bw, i. p. three times a week). The results were as follows: (**A**) Plasma irisin concentrations measured by ELISA (*n* = 4 per group). (**B**) Changes in body weight at 14 days in each group of mice (*n* = 6 per group). (**C**,**D**) Gast and TA muscle morphology mapping and weight analysis (*n* = 6 per group, bars =1 cm). (**E**,**F**) Representative H&E staining and area analysis of Gast muscle fibre cross-sections (bars = 100 μm). (**G**–**I**) Muscle function in mice was analyzed by mouse muscle grip strength, grid suspension capacity and distance to treadmill exhaustion (*n* = 6 per group). (**J**) Western blot analysis of proteins from Atrogin-1, MuRF-1, MSTN, MyHC, myogenin, MyoD, p-mTOR/mTOR, p-P70S6K/P70S6K, p-4E-BP1/4E-BP1, p-AKT/AKT and p-FoxO3a/FoxO3a (*n* = 3–4 per group). (**K**) Puromycin tragic entry analyses of myotubular protein synthesis capacity in groups. Protein band densities were normalized to GAPDH. The data are presented as mean ± SEM; The symbol “•” denotes biological replicates; ^ns^ *p* > 0.05, * *p* < 0.05, ** *p* < 0.01, *** *p* < 0.001.

To further validate the therapeutic efficacy of R-irisin in muscle wasting, we employed a robust glucocorticoid-induced atrophy model using dexamethasone. R-irisin treatment resulted in a significant elevation of circulating irisin levels in both control and dexamethasone-treated mice (Figure 3A). Moreover, a marked attenuation of dexamethasone-induced body weight loss was observed (Figure 3B). Consistent with systemic protection, R-irisin treatment markedly reduced Dex-mediated gastrocnemius and tibialis anterior muscle mass loss (Figure 3C,D). Histological analysis via haematoxylin and eosin (H&E) staining revealed that R-irisin preserved myofibre cross-sectional area in Dex-treated gastrocnemius muscles (Figure 3E,F). Notably, R-irisin treatment also induced a shift towards larger myofibre diameters in control mice (Figure 3E,F), suggesting pro-myogenic effects independent of atrophy reversal. Furthermore, R-irisin therapy improved functional outcomes, evidenced by enhanced grip strength, grid hanging performance, and treadmill exhaustive distance (Figure 3G–I). Collectively, these findings demonstrate that R-irisin exerts potent therapeutic efficacy against Dex-induced muscle atrophy and weakness.

To delineate the molecular mechanisms underlying R-irisin’s anti-atrophic effects, we analyzed the Akt/FoxO signalling pathway and its downstream targets Atrogin-1 and MuRF-1 via Western blotting. Dex treatment reduced Akt and FoxO3a phosphorylation levels, thereby increasing Atrogin-1 and MuRF-1 expression (Figure 3J). Notably, R-irisin administration effectively suppressed Atrogin-1/MuRF-1 upregulation while restoring Akt/FoxO3a phosphorylation in Dex-treated muscles. Strikingly, R-irisin further ameliorated Dex-induced atrophy by elevating myogenic regulatory factors (MyHC, Myogenin, and MyoD) and downregulating the muscle growth inhibitory factor MSTN (Figure 3I). Additionally, R-irisin targets the mTOR signaling pathway and the phosphorylation of its downstream effectors, P70S6K and 4E-BP1—a key regulatory node in the muscle anabolic pathway. (Figure 3J). Functional validation using puromycin incorporation assays in mature myotubes demonstrated that R-irisin significantly enhanced protein synthesis in both control and Dex-treated cells (Figure 3K). These findings collectively reveal that R-irisin counteracts Dex-induced muscle wasting through dual mechanisms: (1) inhibiting catabolic pathways (Atrogin-1/MuRF-1-mediated proteolysis) and (2) stimulating anabolic processes (protein synthesis via mTORC1 activation).

**Figure 4:**
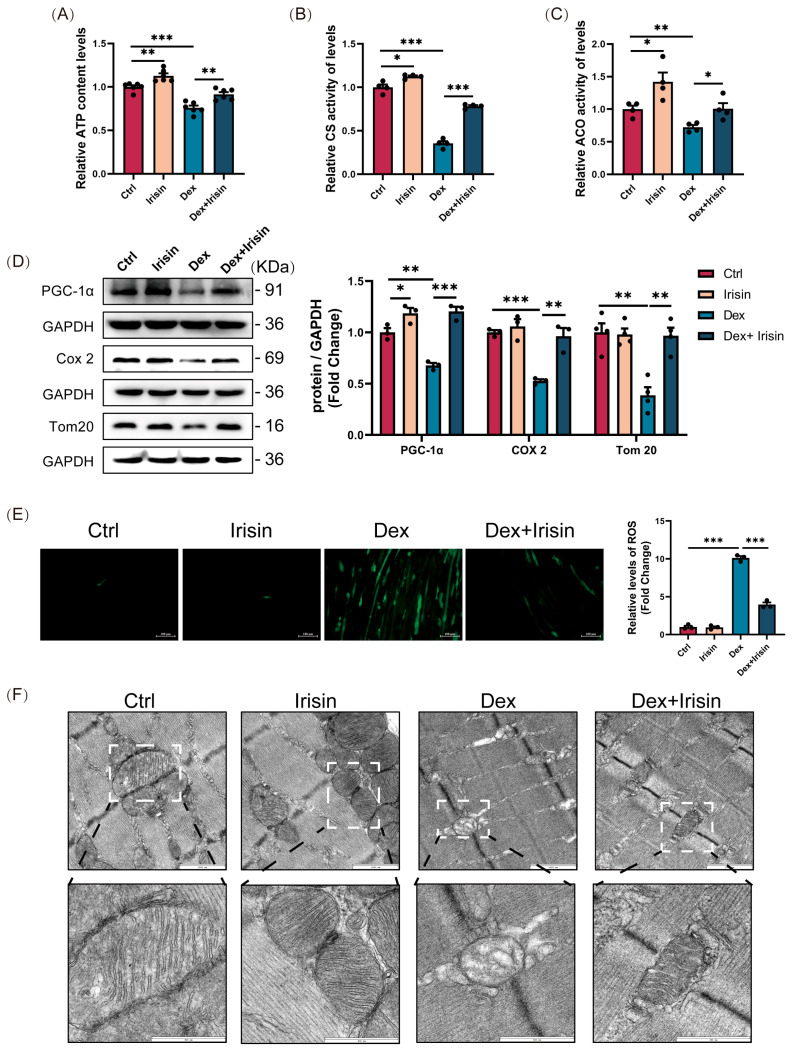
R-irisin reverses Dex-induced mitochondrial dysfunction in mouse skeletal muscle. A murine model of muscle atrophy was induced using a relatively high dose of dexamethasone (25 mg/kg bw, i. p. 14 days) and treated with irisin (2.5 mg/kg bw, i. p. three times a week). The results were as follows: (**A**) Mitochondrial ATP generation capacity in gastrocnemius (Gast) (*n* = 6 per group). (**B**,**C**) Activities of citrate synthase (CS) and aconitase (ACO) in Gast mitochondria (*n* = 4 per group). (**D**) Western blot analysis of mitochondrial biogenesis/function markers PGC-1α, Tom20, and Cox2 in Gast mitochondria (*n* = 3–4 per group). (**E**) Myotube reactive oxygen species (ROS) levels quantified by fluorescence microscopy (scale bar = 100 μm). (**F**) Transmission electron microscopy (TEM) images of Gast mitochondrial morphology (scale bars: 1000 nm/600 nm). All protein band densities have been normalized to the appropriate GAPDH loading control. Results are mean ± SEM; The symbol “•” denotes biological replicates; * *p* < 0.05, ** *p* < 0.01, *** *p* < 0.001.

Muscle atrophy is associated with mitochondrial homeostasis loss, characterized by reduced mitochondrial density (volume), impaired biogenesis, and defective energy production [21]. R-irisin administration ameliorated these defects by enhancing mitochondrial ATP generation, citrate synthase activity, and aconitase activity in Dex-treated muscles (Figure 4A–C). Western blot analyses further confirmed that R-irisin upregulated mitochondrial biogenesis markers PGC-1α, COX2, and TOM20 in Dex-exposed myotubes (Figure 4D). Notably, R-irisin restored redox balance by reducing mitochondrial ROS levels (Figure 4E). These metabolic improvements coincided with structural remodelling of mitochondrial morphology, as evidenced by mitochondrial reduced fragmentation (Figure 4F). Collectively, these findings demonstrate that R-irisin counteracts Dex-induced mitochondrial damage through coordinated enhancement of mitochondrial morphology, biogenesis, and bioenergetic function.

### 3.4. R-Irisin Protects C2C12 Myotubes Against Dex-Induced Atrophy via Sirtuin 1 Activation

**Figure 5:**
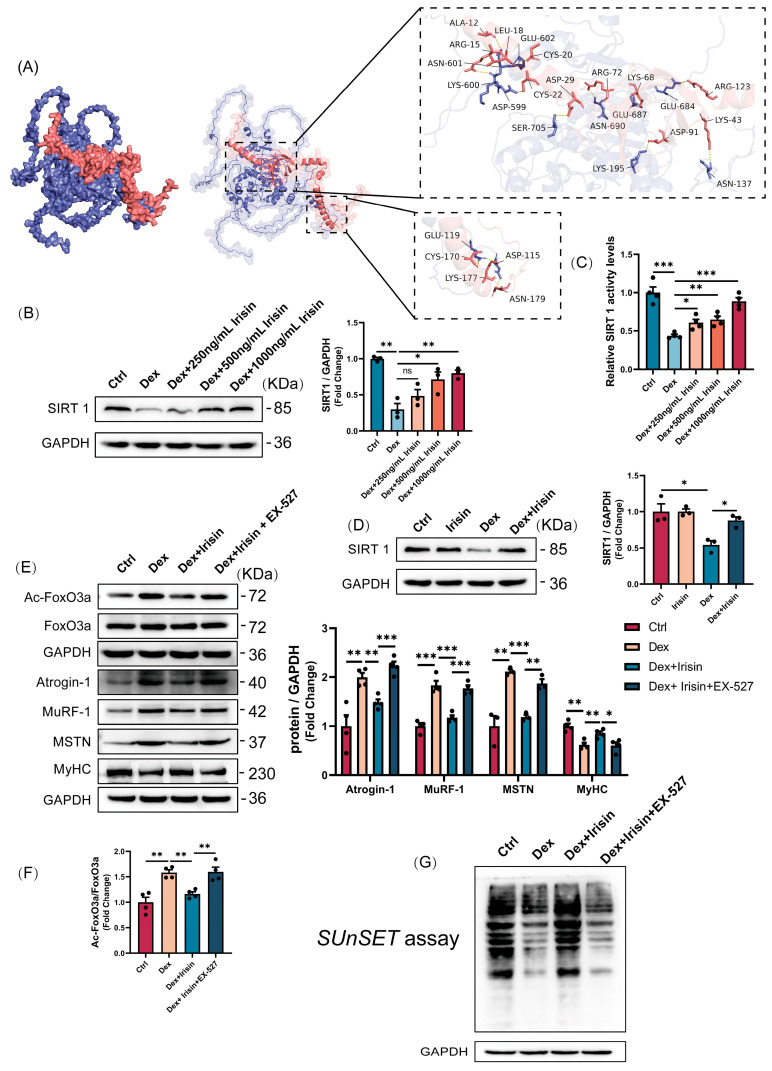
R-irisin protects C2C12 myotubes against Dex-induced atrophy via sirtuin 1 activation. (**A**) Interactive sites between Irisin and SIRT1 by docking analysis. (**B**) Dose-dependent induction of SIRT1 protein expression by irisin in Dex-treated C2C12 myotubes (*n* = 3 per group). (**C**) Enhancement of SIRT1 deacetylase activity by irisin in C2C12 myotubes (*n* = 4 per group). (**D**) Western blot validation of SIRT1 protein levels in gastrocnemius (Gast) (*n* = 3 per group). (**E**,**F**) EX-527 antagonizes irisin-mediated suppression of atrophy markers (Atrogin-1, MuRF-1, MSTN, Ac-FoxO3a/FoxO3a) and induction of myogenic factors (MyHC) in Dex-treated myotubes (*n* = 3–4 per group). (**G**) Puromycin incorporation assays reveal irisin-stimulated protein synthesis in myotubes, which is blocked by EX-527. Protein band densities were normalized to GAPDH. The data are presented as mean ± SEM; The symbol “•” denotes biological replicates; ^ns^ *p* > 0.05, * *p* < 0.05, ** *p* < 0.01, *** *p* < 0.001.

SIRT1 plays a pivotal role in regulating skeletal muscle remodelling. To validate whether irisin directly interacts with SIRT1, we performed virtual docking analysis. The binding affinity of the irisin-SIRT1 complex was calculated to be −12.7 kcal/mol, indicating a strong binding interaction. The detailed interaction interfaces, including key residues involved in the binding, are illustrated in the figure (Figure 5A). Furthermore, treatment with 50 μM dexamethasone significantly reduced SIRT1 levels in C2C12 myotubes (Figure 5B). Strikingly, R-irisin rescued SIRT1 expression in a concentration-dependent manner (Figure 5B). Fluorometric assays further confirmed that R-irisin dose-dependently restored SIRT1 deacetylase activity (Figure 5C). In vivo, R-irisin administration reversed Dex-induced SIRT1 suppression in muscle tissues (Figure 5D). Mechanistically, Dex-mediated SIRT1 inhibition increased FoxO3a acetylation, driving upregulation of atrophy-related proteins atrogin-1, MuRF-1, and MSTN, while suppressing myogenic regulatory factor MyHC. R-irisin restored SIRT1 deacetylase activity, thereby suppressing FoxO3a hyperacetylation and downregulating atrogin-1/MuRF-1/MSTN expression (Figure 5E,F). Notably, co-treatment with the SIRT1-specific inhibitor EX-527 abolished R-irisin’s anti-atrophic effects (Figure 5E,F). Furthermore, R-irisin’s ability to rescue Dex-induced deficits in protein synthesis—assessed via puromycin incorporation assays—was dependent on SIRT1 activity (Figure 5G). These findings establish that R-irisin exerts its protective effects through SIRT1-mediated deacetylation pathways, coordinately regulating proteostasis and myogenesis.

### 3.5. R-Irisin Protects C2C12 Myotubes Against Dex-Induced Mitochondrial Dysfunction via Sirtuin 1 Activation

**Figure 6:**
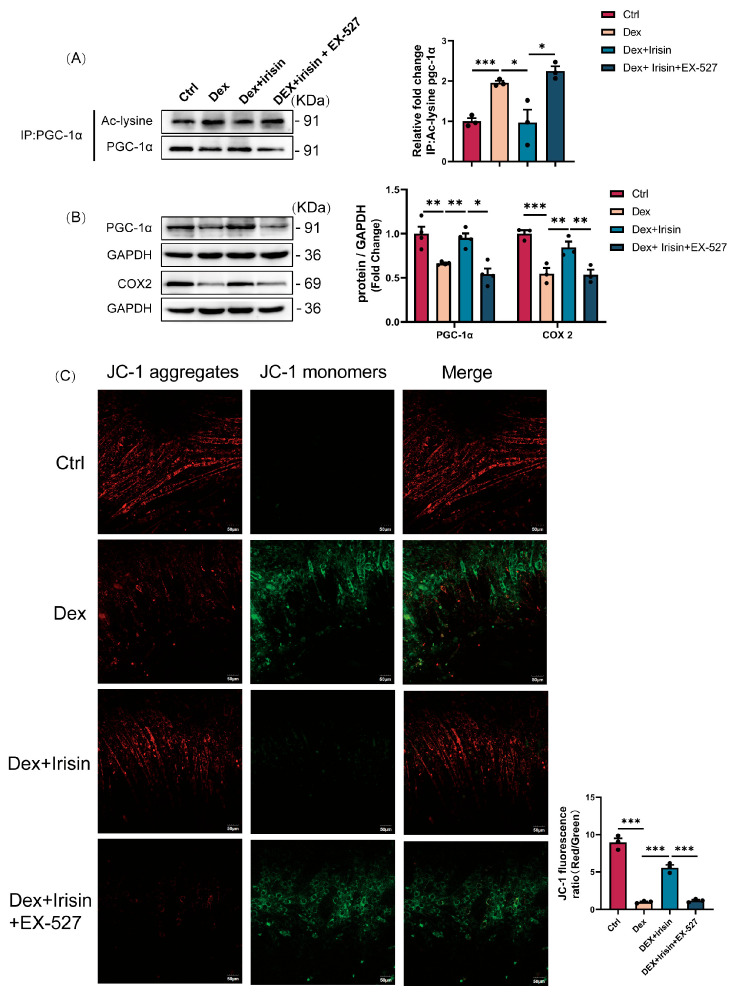
R-irisin attenuates Dex-induced mitochondrial dysfunction in C2C12 myotubes via SIRT1 activation. (**A**,**B**) Western blot validation of mitochondrial function markers: acetylated PGC-1α (Ac-PGC-1α), total PGC-1α, and COX2 in C2C12 myotubes (*n* = 3–4 per group). (**C**) JC mitochondrial membrane potential assay quantification (bars = 50 μm; *n* = 3 per group). Protein band densities were normalized to GAPDH. The data are presented as mean ± SEM; The symbol “•” denotes biological replicates; * *p* < 0.05, ** *p* < 0.01, *** *p* < 0.001.

PGC-1α, a key substrate of SIRT1 deacetylation and its biologically active form ^13^, was significantly hyperacetylated in Dex-treated C2C12 myotubes. Immunoprecipitation (IP) revealed that R-irisin treatment reversed Dex-induced elevation of acetylated PGC-1α (Figure 6A). Consistent with these findings, co-administration of the SIRT1 inhibitor EX-527 abolished R-irisin’s effects on both total and acetylated PGC-1α levels (Figure 6A). Western blot analyses further demonstrated that R-irisin-mediated restoration of mitochondrial biogenesis markers and function was blocked by EX-527 (Figure 6B). Mitochondrial membrane potential staining showed that Dex induced a collapse in membrane potential, which was rescued by R-irisin but reversed by EX-527 co-treatment (Figure 6C). Collectively, these results establish that R-irisin alleviates Dex-induced atrophy and mitochondrial dysfunction by restoring SIRT1-dependent deacetylation of FoxO3a and PGC-1α, thereby reactivating their pro-myogenic and bioenergetic functions.

### 3.6. R-Irisin Protects Mice Against Dex-Induced Muscle Atrophy and Dysfunction via SIRT1 Targeting

**Figure 7:**
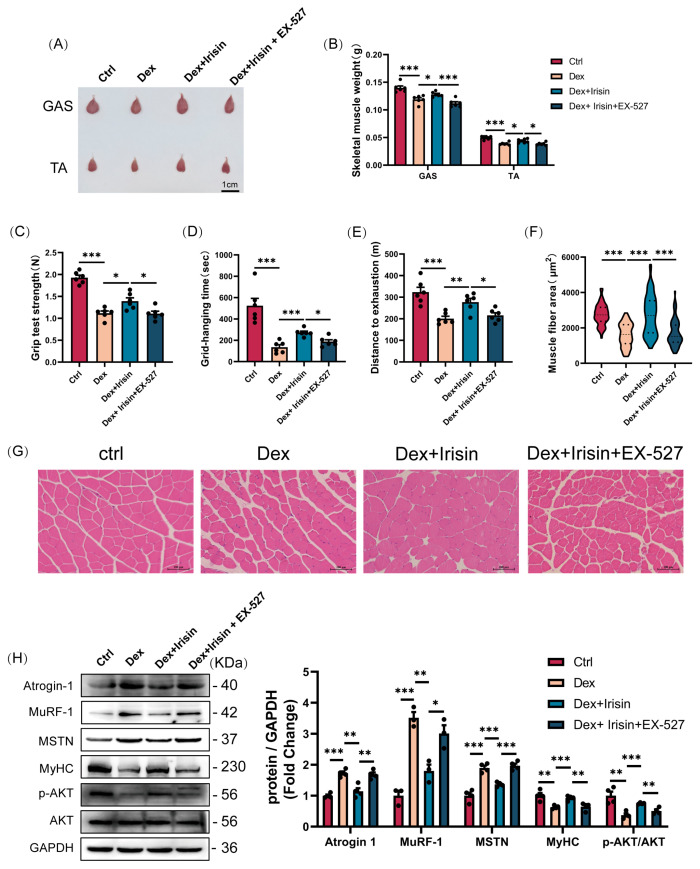
R-irisin attenuates Dex-induced muscle atrophy in mice via SIRT1 activation. To evaluate the interplay between irisin and SIRT1 signaling in Dex-induced muscle atrophy, we implemented a combined treatment regimen: irisin was co-administered with the SIRT1 inhibitor EX-527 (10 mg/kg body weight, i. p. 14 days). The results were as follows: (**A**,**B**) Gastrocnemius (Gast) and tibialis anterior (TA) muscle morphology and mass analysis (*n* = 6 per group). (**C**–**E**) Functional assessment of muscle grip strength, grid hanging capacity, and treadmill exhaustive distance in mice (*n* = 6 per group). (**F**,**G**) Histological analysis of Gast myofibre cross-sectional area (CSA) via H&E staining (scale bars = 100 μm; *n* = 6 per group). (**H**) Western blot quantification of atrophy markers (atrogin-1, MuRF-1, MSTN) and myogenic regulators (MyHC, p-AKT/AKT) (*n* = 3–4 per group). Protein band densities were normalized to GAPDH. The data are presented as mean ± SEM; The symbol “•” denotes biological replicates; * *p* < 0.05, ** *p* < 0.01, *** *p* < 0.001.

**Figure 8:**
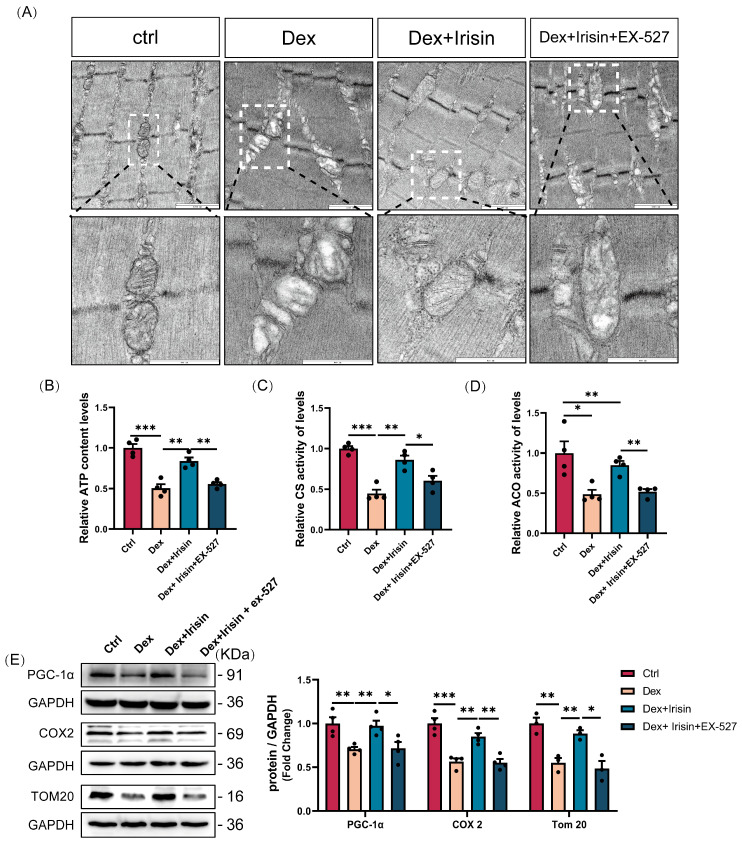
R-irisin attenuates Dex-induced muscle mitochondrial dysfunction in mice via SIRT1 activation. To evaluate the interplay between irisin and SIRT1 signaling in Dex-induced muscle atrophy, we implemented a combined treatment regimen: irisin was co-administered with the SIRT1 inhibitor EX-527 (10 mg/kg body weight, i. p. 14 days). (**A**) Transmission electron microscopy (TEM) analysis of mitochondrial morphology in gastrocnemius (Gast) (bars = 1000 nm/600 nm; *n* = 3 per group). (**B**) ATP generation capacity in Gast mitochondria (*n* = 4 per group). (**C**,**D**) Enzymatic activities of citrate synthase (CS) and aconitase (ACO) in Gast mitochondria (*n* = 4 per group). (**E**) Western blot quantification of mitochondrial biogenesis/function markers PGC-1α, Tom20, and COX2 in Gast (*n* = 3–4 per group). Protein band densities were normalized to GAPDH. The data are presented as mean ± SEM; The symbol “•” denotes biological replicates; * *p* < 0.05, ** *p* < 0.01, *** *p* < 0.001.

Building on R-irisin’s robust in vitro efficacy, we extended our investigation to in vivo models. R-irisin administration effectively restored Dex-induced losses in gastrocnemius and tibialis anterior muscle mass, while co-administration with the SIRT1 inhibitor EX-527 antagonized these therapeutic effects (Figure 7A,B). Similarly, R-irisin-enhanced muscle functional performance-including grip strength, grid hanging capacity, and treadmill exhaustive distance-was nearly abolished by EX-527 co-treatment (Figure 7C–E). Histological analysis revealed that R-irisin prevented Dex-induced reductions in myofibre cross-sectional area (Figure 7F,G) and suppressed expression of key atrophy-related proteins: Atrogin-1, MuRF-1, and MSTN (Figure 7H), effects that were reversed by EX-527. Notably, R-irisin rescued Dex-mediated mitochondrial dysfunction, evidenced by restored mitochondrial integrity (Figure 8A), enhanced ATP concentration, citrate synthase activity, and aconitase activity (Figure 8B–D). These mitochondrial improvements coincided with upregulated biogenesis markers PGC-1α, COX2, and Tom20 (Figure 8E), all of which were antagonized by EX-527. Collectively, these in vivo findings confirm that R-irisin mitigates Dex-induced muscle atrophy and mitochondrial dysfunction through SIRT1-dependent pathways, reinforcing its therapeutic potential for sarcopenic disorders.

## 4. Discussion

Sarcopenia, characterized by progressive skeletal muscle mass and strength loss, significantly impairs mobility, quality of life, and healthcare systems due to fall-related injuries, costly hospitalisations, and prolonged rehabilitation [22]. Despite the established association with aging [23], current therapeutic paradigms remain suboptimal. While resistance exercise remains the cornerstone intervention, its applicability is severely limited in sarcopenic populations with functional impairments. Pharmacological strategies targeting anabolic hormones (e.g., testosterone, growth hormone) exhibit inconsistent efficacy profiles, dose-limiting adverse effects, and marginal functional improvements [24], underscoring the imperative for mechanism-driven therapeutic innovations.

**Figure 9:**
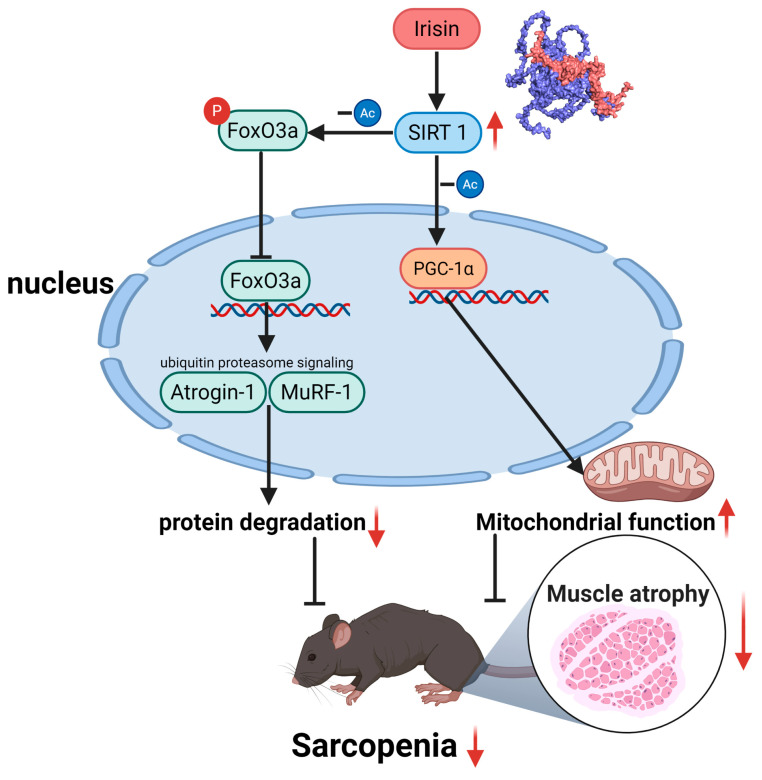
A SIRT1-dependent axis that synergistically suppresses catabolic pathways while enhancing anabolic signaling.

Our discovery of irisin’s dual regulatory role in proteostasis and mitochondrial homeostasis provides novel mechanistic insights into its therapeutic potential. The observed downregulation of circulating irisin in glucocorticoid-induced murine models, establishes a compelling correlative link between irisin deficiency and muscle wasting pathophysiology. Mechanistically, we delineate a SIRT1-dependent axis that synergistically suppresses catabolic pathways while enhancing anabolic signaling (Figure 9). This regulatory cascade positions irisin as a master coordinator of skeletal muscle plasticity, bridging exercise mimetic effects with intracellular metabolic regulation.

The circulating levels of irisin have been identified as a biomarker for muscle mass and performance [25]. For instance, aging [26] and denervation-induced [17] skeletal muscle loss and atrophy are associated with reduced irisin concentrations, potentially implicating irisin deficiency as a contributing factor to muscle wasting. In glucocorticoid-induced muscle atrophy, we observed a significant downregulation of FNDC5/irisin levels both in the circulation of mice in vivo and in ex vivo cultured C2C12 myotubes. This consistent reduction strongly suggests that irisin deficiency is a pathogenic factor driving glucocorticoid-induced muscle atrophy. Beyond its well-documented endocrine and paracrine roles in metabolic organs [27], irisin exerts autocrine effects within muscle tissue. It upregulates growth-related genes in myocytes [28], while enhancing myoblast proliferation and fusion [17], thereby promoting muscle growth and rescuing dystrophic phenotypes in murine models of Duchenne muscular dystrophy [29], denervation [17], and hindlimb suspension [30]. Our findings extend irisin’s therapeutic relevance to glucocorticoid-associated sarcopenia. Here, we demonstrate that irisin simultaneously promotes myotube development by upregulating MyHC, MyoD, and MyoG expression while suppressing atrophy-related proteins Atrogin-1, MuRF-1, and MSTN to counteract glucocorticoid-induced muscle wasting. Given irisin’s classification as an exercise mimetic [31], we investigated its capacity to induce exercise-like phenotypes in murine muscle. Strikingly, irisin enhanced muscle strength in both healthy and atrophied murine models, evidenced by improved grip strength, grid-hanging performance, and increased treadmill endurance capacity. Subsequent cross-sectional area analysis revealed marked hypertrophic phenotypes in irisin-treated muscle tissues. Collectively, these observations align with characteristic adaptations induced by resistance exercise training.

SIRT1 critically regulates skeletal muscle remodelling through deacetylation-mediated modulation of FoxO transcriptional activity, which governs muscle mass and function [32]. Here, we demonstrate that irisin enhances SIRT1 protein expression and deacetylase activity in a dose-dependent manner, thereby shifting the FoxO3a phosphorylation-acetylation equilibrium to suppress its nuclear translocation. This molecular mechanism provides a mechanistic basis for irisin-mediated inhibition of Atrogin-1 and MuRF1 expression. The Akt/mTOR axis, a central signalling pathway for skeletal muscle protein synthesis [33], is restored by irisin to counteract glucocorticoid-induced protein synthesis defects. Puromycin incorporation assays revealed that irisin also enhances protein synthesis in normal myotubes, suggesting a broader anabolic role in muscle hypertrophy. Notably, irisin’s dual modulation of Akt/mTOR (enhancing translation initiation) and ubiquitin-proteasome system (reducing proteolysis) mirrors the physiological biphasic response to mechanical loading, wherein anabolic and anti-catabolic pathways are temporally segregated during muscle repair. This mechanistic duality positions irisin as a superior alternative to current therapies targeting single pathways, particularly in glucocorticoid-induced myopathy where dual-pathway dysregulation predominates.

Beyond its role in proteostasis, irisin exerts profound effects on mitochondrial biogenesis and function, which are critical in glucocorticoid-induced muscle atrophy. PGC-1α, a master regulator of mitochondrial biogenesis and cellular energy metabolism, drives mitochondrial biogenesis by stimulating transcription factors nuclear respiratory factor 1 and 2 (NRF-1 and NRF-2), thereby increasing mitochondrial enzyme transcription [34]. During muscle atrophy, mitochondrial degradation reduces mitochondrial mass and quantity, governed by mitophagy and mitochondrial fusion/fission dynamics [35]. These mitochondrial quality control systems are essential for maintaining skeletal muscle mass by identifying and correcting mitochondrial dysfunction. Mitochondrial dysfunction triggers catabolic pathways that feedback to the nucleus to promote expression of muscle atrophy-related genes [36]. Here, irisin enhances PGC-1α expression and reduces its acetylation status via SIRT1 activation, augmenting its transcriptional activity. This results in significant improvements in mitochondrial DNA synthesis, ATP production, and enzymatic activities of citrate synthase and aconitase, collectively restoring mitochondrial morphology and function. Normalization of mitochondrial parameters correlates with functional recovery, evidenced by improved grip strength and treadmill endurance in treated mice. This mitochondrial remodelling effect positions irisin as a central regulator of systemic energy homeostasis. By simultaneously enhancing mitochondrial biogenesis and protein synthesis, irisin creates a synergistic anabolic environment. This dual mechanism is particularly relevant in aged populations, where mitochondrial dysfunction serves as a primary driver of sarcopenia progression.

## 5. Conclusions

Collectively, our findings position irisin as a critical mediator of exercise-induced benefits, counteracting glucocorticoid-driven muscle wasting through SIRT1-dependent regulation of proteostasis and mitochondrial homeostasis. These results pave the way for developing irisin-based therapies-either through exogenous supplementation or endogenous activation-as practical alternatives to exercise for sarcopenia management.

## Data Availability

The original contributions presented in this study are included in the article/Supplementary Materials. Further inquiries can be directed to the corresponding author.

## Supplementary Materials

The following supporting information can be downloaded at https://www.mdpi.com/article/10.3390/cells14211675/s1: Table S1. Antibody for Western Blot.

## References

[1] Thyfault J.P., Bergouignan A. (2020). Exercise and metabolic health: Beyond skeletal muscle. Diabetologia.

[2] Cohen S., Nathan J.A., Goldberg A.L. (2014). Muscle wasting in disease: Molecular mechanisms and promising therapies. Nat. Rev. Drug Discov..

[3] Creus A., Sebastián D. (2024). Age-related muscle atrophy? Mitofusin 2 the rescue. Acta Physiol..

[4] Rhen T., Cidlowski J.A. (2005). Antiinflammatory Action of Glucocorticoids—New Mechanisms for Old Drugs. N. Engl. J. Med..

[5] Schakman O., Kalista S., Barbé C., Loumaye A., Thissen J. (2013). Glucocorticoid-induced skeletal muscle atrophy. Int. J. Biochem. Cell Biol..

[6] Braun T.P., Marks D.L. (2015). The regulation of muscle mass by endogenous glucocorticoids. Front. Physiol..

[7] Batchelor T.T., Taylor L.P., Thaler H.T., Posner J.B., DeAngelis L.M. (1997). Steroid myopathy in cancer patients. Neurology.

[8] Latres E., Amini A.R., Amini A.A., Griffiths J., Martin F.J., Wei Y., Lin H.C., Yancopoulos G.D., Glass D.J. (2005). Insulin-like Growth Factor-1 (IGF-1) Inversely Regulates Atrophy-induced Genes via the Phosphatidylinositol 3-Kinase/Akt/Mammalian Target of Rapamycin (PI3K/Akt/mTOR) Pathway. J. Biol. Chem..

[9] Gumucio J.P., Mendias C.L. (2012). Atrogin-1, MuRF-1, and sarcopenia. Endocrine.

[10] Romanello V., Sandri M. (2013). Mitochondrial biogenesis and fragmentation as regulators of protein degradation in striated muscles. J. Mol. Cell. Cardiol..

[11] Ruas J.L., White J.P., Rao R.R., Kleiner S., Brannan K.T., Harrison B.C., Greene N.P., Wu J., Estall J.L., Irving B.A. (2012). A PGC-1α Isoform Induced by Resistance Training Regulates Skeletal Muscle Hypertrophy. Cell.

[12] Lin S., Xing H., Zang T., Ruan X., Wo L., He M. (2018). Sirtuins in mitochondrial stress: Indispensable helpers behind the scenes. Ageing Res. Rev..

[13] Gurd B.J. (2011). Deacetylation of PGC-1α by SIRT1: Importance for skeletal muscle function and exercise-induced mitochondrial biogenesis. Appl. Physiol. Nutr. Metab..

[14] Fan W., Evans R.M. (2017). Exercise Mimetics: Impact on Health and Performance. Cell Metab..

[15] Boström P., Wu J., Jedrychowski M.P., Korde A., Ye L., Lo J.C., Rasbach K.A., Boström E.A., Choi J.H., Long J.Z. (2012). A PGC1-α-dependent myokine that drives brown-fat-like development of white fat and thermogenesis. Nature.

[16] Chang J.S., Kong I.D. (2020). Irisin prevents dexamethasone-induced atrophy in C2C12 myotubes. Pflug. Arch. Eur. J. Physiol..

[17] Reza M.M., Subramaniyam N., Sim C.M., Ge X., Sathiakumar D., McFarlane C., Sharma M., Kambadur R. (2017). Irisin is a pro-myogenic factor that induces skeletal muscle hypertrophy and rescues denervation-induced atrophy. Nat. Commun..

[18] Vinel C., Lukjanenko L., Batut A., Deleruyelle S., Pradère J.-P., Le Gonidec S., Dortignac A., Geoffre N., Pereira O., Karaz S. (2018). The exerkine apelin reverses age-associated sarcopenia. Nat. Med..

[19] Cai Q., Sahu R., Ueberschlag-Pitiot V., Souali-Crespo S., Charvet C., Silem I., Cottard F., Ye T., Taleb F., Metzger E. (2024). LSD1 inhibition circumvents glucocorticoid-induced muscle wasting of male mice. Nat. Commun..

[20] Yang J.-C., Liu M., Huang R.-H., Zhao L., Niu Q.-J., Xu Z.-J., Wei J.-T., Lei X.G., Sun L.-H. (2024). Loss of SELENOW aggravates muscle loss with regulation of protein synthesis and the ubiquitin-proteasome system. Sci. Adv..

[21] Romanello V., Sandri M. (2010). Mitochondrial Biogenesis and Fragmentation as Regulators of Muscle Protein Degradation. Curr. Hypertens. Rep..

[22] Larsson L., Degens H., Li M., Salviati L., Lee Y.I., Thompson W., Kirkland J.L., Sandri M. (2019). Sarcopenia: Aging-Related Loss of Muscle Mass and Function. Physiol. Rev..

[23] Martinez B.P., Batista A.K.M.S., Gomes I.B., Olivieri F.M., Camelier F.W.R., Camelier A.A. (2015). Frequency of sarcopenia and associated factors among hospitalized elderly patients. BMC Musculoskelet. Disord..

[24] Giannoulis M.G., Martin F.C., Nair K.S., Umpleby A.M., Sonksen P. (2012). Hormone Replacement Therapy and Physical Function in Healthy Older Men. Time to Talk Hormones?. Endocr. Rev..

[25] Chang J.S., Kim T.H., Nguyen T.T., Park K., Kim N., Kong I.D. (2017). Circulating irisin levels as a predictive biomarker for sarcopenia: A cross-sectional community-based study. Geriatr. Gerontol. Int..

[26] Guo M., Yao J., Li J., Zhang J., Wang D., Zuo H., Zhang Y., Xu B., Zhong Y., Shen F. (2022). Irisin ameliorates age-associated sarcopenia and metabolic dysfunction. J. Cachex-Sarcopenia Muscle.

[27] Shi H., Hao X., Sun Y., Zhao Y., Wang Y., Cao X., Gong Z., Ji S., Lu J., Yan Y. (2024). Exercise-inducible circulating extracellular vesicle irisin promotes browning and the thermogenic program in white adipose tissue. Acta Physiol..

[28] Huh J.Y., Dincer F., Mesfum E., Mantzoros C.S. (2014). Irisin stimulates muscle growth-related genes and regulates adipocyte differentiation and metabolism in humans. Int. J. Obes..

[29] Reza M.M., Sim C.M., Subramaniyam N., Ge X., Sharma M., Kambadur R., McFarlane C. (2017). Irisin treatment improves healing of dystrophic skeletal muscle. Oncotarget.

[30] Colaianni G., Mongelli T., Cuscito C., Pignataro P., Lippo L., Spiro G., Notarnicola A., Severi I., Passeri G., Mori G. (2017). Irisin prevents and restores bone loss and muscle atrophy in hind-limb suspended mice. Sci. Rep..

[31] Zhang H., Wu X., Liang J., Kirberger M., Chen N. (2022). Irisin, an exercise-induced bioactive peptide beneficial for health promotion during aging process. Ageing Res. Rev..

[32] Lee D., Goldberg A.L. (2013). SIRT1 Protein, by Blocking the Activities of Transcription Factors FoxO_1_ and FoxO_3_, Inhibits Muscle Atrophy and Promotes Muscle Growth. J. Biol. Chem..

[33] Dreyer H.C., Fujita S., Glynn E.L., Drummond M.J., Volpi E., Rasmussen B. (2010). Resistance exercise increases leg muscle protein synthesis and mTOR signalling independent of sex. Acta Physiol..

[34] Jäger S., Handschin C., St.-Pierre J., Spiegelman B.M. (2007). AMP-activated protein kinase (AMPK) action in skeletal muscle via direct phosphorylation of PGC-1alpha. Proc. Natl. Acad. Sci. USA.

[35] Sakellariou G.K., Pearson T., Lightfoot A., Nye G., Wells N., Giakoumaki I.I., Vasilaki A., Griffiths R.D., Jackson M., McArdle A. (2016). Mitochondrial ROS regulate oxidative damage and mitophagy but not age-related muscle fiber atrophy. Sci. Rep..

[36] Romanello V., Sandri M. (2020). The connection between the dynamic remodeling of the mitochondrial network and the regulation of muscle mass. Cell. Mol. Life Sci..

