# Supplementary material for: Irisin Increases Sirtuin 1 to Improve Glucocorticoid-Induced Sarcopenia and Mitochondrial Dysfunction
Source: Cells. 2025 Oct 27;14(21):1675. doi: 10.3390/cells14211675 (PMC12607365; doi:10.3390/cells14211675)
Supplement: Supplementary file 1 [file cells-14-01675-s001.zip › cells-3889581-supplementary.pdf]

Supplementary Table S1. Antibody for Western Blot

Antisera	Source	Antibody Cat. No.	Dilution
Atrogin-1	Proteintech	67172-1-Ig	1:5000
MuRF-1	Proteintech	55456-1-AP	1:5000
MSTN	Proteintech	19142-1-AP	1:5000
MyHC	Proteintech	22287-1-AP	1:1000
mTOR	Proteintech	66888-1-Ig	1:1000
Phospho-mTOR (Ser2448)	Proteintech	67778-1-Ig	1:1000
4E-BP1	Immunoway	YM8369	1:2000
Phospho-4E-BP1 (Thr70)	Immunoway	PT1203R	1:2000
p70S6k	Proteintech	14485-1-AP	1:2000
Phospho-p70S6k (Ser371)	Immunoway	YM8688	1:2000
FoxO3a	Proteintech	10849-1-AP	1:1000
phospho-FoxO3a(Ser294)	Immunoway	YP1338	1:1000
Acetyl-FoxO3a	Immunoway	YK0112	1:1000
Akt	Cell Signaling	9272S	1:1000
Phospho-Akt (Ser473)	Cell Signaling	4060S	1:1000
Tom20	Immunoway	YM8164	1:1000
Cox2	Proteintech	Cox2	1:1000
PGC-1α	Proteintech	66369-1-Ig	1:1000
Bax	Proteintech	50599-2-Ig	1:1000
Bcl-2	Proteintech	26593-1-AP	1:1000
FNDC5	Abcam	ab131390	1:1000
Myogenin	Abcam	ab103924	1:1000
MyoD	Abcam	ab64159	1:1000
SIRT1	Santa Cruz	sc-74465	1:500
Puromycin	Millipore	MABE343	1:1000
Ac-lysine	Cell Signaling	9681T	1:1000
GAPDH	Proteintech	60004-1-Ig	1:10000
